# Surprising acidity for the methylene of 1,3-indenocorannulenes?

**DOI:** 10.3762/bjoc.20.260

**Published:** 2024-12-02

**Authors:** Shi Liu, Märt Lõkov, Sofja Tshepelevitsh, Ivo Leito, Kim K Baldridge, Jay S Siegel

**Affiliations:** 1 School of Pharmaceutical Science and Technology, Tianjin University, Tianjin, PR China; 2 University of Tartu, Institute of Chemistry, Ravila 14a, Tartu 50411, Estonia

**Keywords:** acidity, aromatic hydrocarbon, Clar sextet, Loschmidt group element, molecular graph

## Abstract

Quantitative assessment of the first acidity constant (p*K*_a_) for BFC (27.6 in CH_3_CN) and FIC (27.8 in CH_3_CN) shows the methylene protons to be significantly more acidic than those in related cyclopentadiene (32 in CH_3_CN), indene (34 in CH_3_CN), or fluorene (37 in CH_3_CN) and comparable to the methine of 9-perfluorophenylfluorene (28.14 in CH_3_CN). This work reports quantitative p*K*_a_ values of BFC and FIC, places those values in a broadened context of CpH-cognate hydrocarbon acidity and presents a Clar–Loschmidt graph perspective to help understand the “surprises”.

## Introduction

**Scheme 1:**
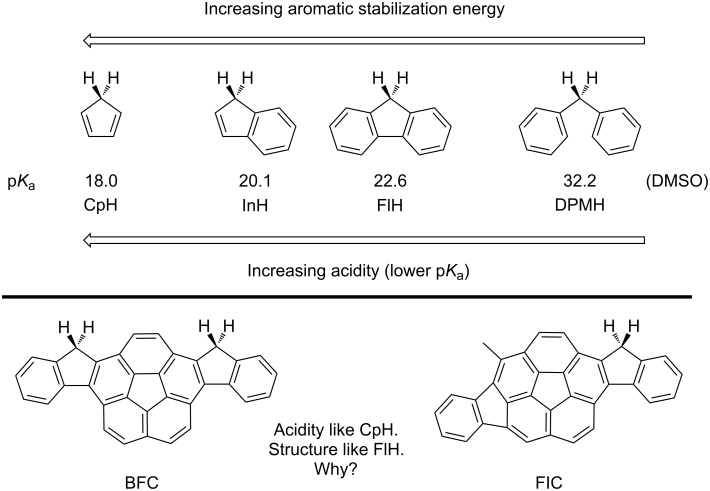
Aromatic stabilization energy across a series of small aromatics (upper); graphical depiction of the BFC/FIC acidity quandry (lower).

A classic textbook tetrad linking hydrocarbon acidity to aromatic stabilization energy comprises cyclopentadiene (CpH), indene (InH), fluorene (FlH), and diphenylmethane (DPMH) [1–2], with p*K*_a_ values in DMSO equal to 18 [3], 20.1 [3], 22.6 [3], and 32.2 [4], respectively (Scheme 1) [5–6]. The reaction enthalpy for deprotonation of CpH is ca 20 kcal/mol less endothermic than DPMH and ca. 24–27 kcal/mol less endothermic than 1,4-pentadiene (PDH; p*K*_a_ ≈ 35 in DMSO) [3], values strikingly similar to the resonance stabilization energy estimated for benzene [7–8]. Furthermore, the trend of p*K*_a_ values for CpH, InH, and FlH correlates with the reduction of the aromatic stabilization energy for the anion across the series [1–2]. At first glance, this model supports the notion that the relative p*K*_a_ values of cyclopentadienes embedded in polynuclear aromatic hydrocarbons (CpH-PAHs) reflect some general measure of the embedded CpH's aromatic character. In this context, the observation that BFC and FIC (FlH cognates) manifest acidity comparable to CpH, was surprising [9]. This study uses a Clar–Loschmidt graph model to show that the acidity of CpH-PAH is more nuanced [10–11]. Subsequently, literature and computational examples reveal a leitmotif for CpH-PAH-based redox-active carbon-rich materials.

## Results and Discussion

From competitive titrations against 9-C_6_F_5_-fluorene (p*K*_a_ = 28.14 in CH_3_CN) [12–13] and (4-Me-C_6_F_4_)(C_6_H_5_)CHCN (p*K*_a_ = 26.98 in CH_3_CN) [12–13] as upper and lower references, respectively, the acidities of BFC (p*K*_a_ = 27.6 in CH_3_CN) and FIC (p*K*_a_ = 27.8 in CH_3_CN) could be quantified and ordered relative to a series of organic aromatic acids (Table 1). Rather than falling among the series cyclopentadiene (p*K*_a_ = 32 in CH_3_CN), indene (p*K*_a_ = 34 in CH_3_CN), or fluorene (p*K*_a_ = 37 in CH_3_CN), BFC and FIC rank as stronger acids than CpH in acetonitrile. Extrapolation to DMSO by conversion equations, supports the assertion that BFC and FIC are more acidic than CpH in an “absolute acidity” sense [13].

**Table 1:** Relative aromatic hydrocarbon acidities I.

Compound	CH_3_CN	CH_3_CN	DMSO	DMSO
	Cmptl^a^	Exptl/Est^b^	Cmptl^a^	Exptl/Est^b^

fluorene	35.0	37^b,c^	21.6	22.6^d^
indene	32.2	34^b,c^	18.8	20.1^d^
cyclopentadiene	29.1	32^b,c^	15.6	18.0^d^
FIC	27.8	27.8^e^	14.4	14.4^b,e^
BFC	28.3	27.6^e^	14.3	14.3^b,e^
9-phenylfluorene	30.8	32^b,c^	17.4	17.9^f^
fluoradene	22.8	23.9^b,c^	9.4	10.5^f^
diphenylmethane	43.4	48^b,c^	29.9	32.2^g^

^a^B97-D/def2-TZVPP(solvent)//B97-D/def2-TZVPP(solvent) referenced to 9-C_6_F_5_-fluorene (solvent). ^b^Estimated using experimental data and correlation equations. ^c^Ref. [13]. ^d^Ref. [3]. ^e^This work. ^f^Ref. [5]. ^g^Ref. [4].

How odd is such a p*K*_a_ value of ca. 14 for a CpH-PAH like BFC or FIC in DMSO? From the initial structural analogy to fluorene there is a substantial 8 p*K*_a_ unit difference, which is nearly 11 kcal/mol in ambient reaction free energy. Computations (B97-D/def2-TZVPP(THF)//B97-D/def2-TZVPP(THF)) also predict p*K*_a_ values of 14.4 and 14.3 for FIC and BFC in DMSO, respectively, so to the extent that these values are surprising, the problem must mostly come from a distortion of our perspective, and not from any dispute between theory and experiment. If that is the case, then what might be a better perspective to investigate CpH-PAH structures like BIC and FIC?

Let us consider a perspective based on the teachings of Clar and Loschmidt. Clar held the electron sextet as the paragon of aromaticity [14] and Loschmidt considered benzene a "group element" [15]. Combining these perspectives, one can view PAH networks as graphs comprised of Clar–Loschmidt (CL) "elements" [10,16]. For corannulene, a maximum of two CL elements (sextets) are present in any one resonance form. Therefore, applying the CL perspective to BFC and FIC one sees dibenzofluorene (full name 13*H*-dibenzo[*a*,*c*]fluorene) as a cognate, which highlights these compounds to be like a conformationally planar-locked 2,3-diphenylindene (p*K*_a_ 17.7 in DMSO [17].

**Scheme 2:**
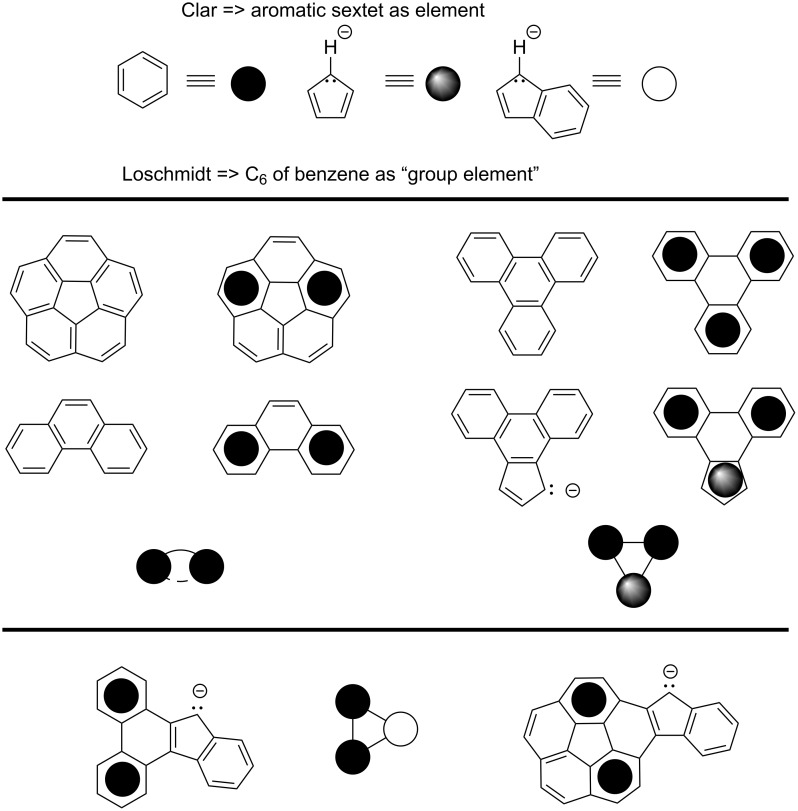
Clar–Loschmidt graphs: [upper] defining the relationship of the molecular fragment to the graph node (shaded circle) for benzene, Cp anion, and indene anion; [middle], superposition of the graph node onto the molecular fragment and then representation of the nodes in the abstract graph: corannulene above phenanthrene (left); triphenylene above cyclopentaphenanthrene (right); [lower] as with middle but dibenzofluorene (left) and fluorenocorannulene (right).

The CL perspective also allows one to create graphs with points representing the aromatic elements, such as, benzene, Cp anion, pyrrole, etc. (Scheme 2, top). Triphenylene is a simple cyclic graph of three benzene elements and their direct connections. A simple modification of the triphenylene graph is the replacement of benzene by Cp anion (Scheme 2, middle). From the CL graph perspective, the anion of cyclopentaphenanthrene is related to that of dibenzofluorene the same way as the Cp anion is related to that of indene. Therefore, cyclopentaphenanthrene should be more acidic than dibenzofluorene, which it is [18]. Monofluorenocorannulene and dibenzofluorene map onto a common CL graph with benzene and indenyl elements (Scheme 2, bottom). These compounds display related electronic structures and are predicted computationally to have similar p*K*_a_ values (i.e., show similar acidity).

**Scheme 3:**
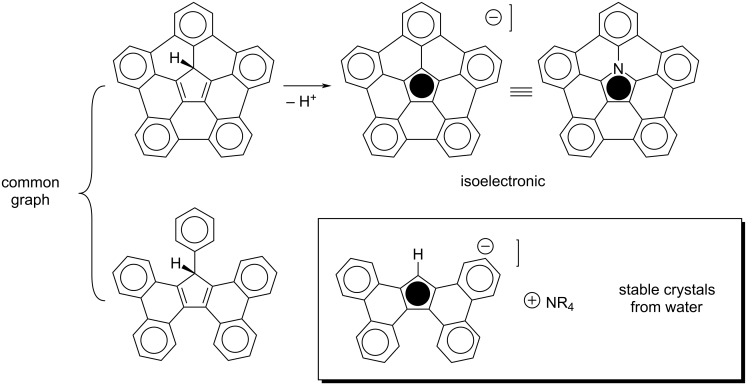
CL graph perspective on acidic PAH-CpHs; pentabenzocorannulene and pentabenzoazocorannulene (upper); phenyltetrabenzofluorene and tetrabenzofluorene TBF (lower).

The extreme of this graph example is the five rim benzene elements and a Cp^−^ core, pentabenzocorannulene (Scheme 3, upper) [19–20]. Such a CpH derivative should be a very acidic aromatic hydrocarbon, and computations for this study predict its p*K*_a_ to be ca 1.1 in DMSO. This high acidity is well seen if one recognizes that, when seen through the CL model, pentabenzocorannulene is more of a well-overlapping pentaphenyl-CpH derivative than a pentabenzocorannulene. Swap out Cp^−^ by the isolelectronic pyrrole and it becomes clear that pentabenzoazacorannulene is also less of an azocorannulene and more a pentaphenylpyrrole, which explains its ease of synthesis and the physical properties [21–22].

The graph with four benzene elements and a CpH core is evident in the commercially available tetrabenzofluorene, TBF (Scheme 3, lower). The parent and the phenyl TBF derivative were also recently prepared by a mechanochemical Scholl reaction [23–25]. Furthermore, the anion of TBF is stable enough to be easily handled and the crystal structures of a number of common ammonium salts of TBF anion, grown from water, have been reported [26].

Neutral highly acidic compounds tend to deprotonate to form highly stable anions. The air stability of TBF conforms to that model. In contrast, although the dianion of BFC can be generated with *tert*-butoxide under inert atmosphere, in the presence of water it quenches and exposed to oxygen it oxidizes to form the diketone. Access to the dianion of BFC presages an interesting diradical and this was achieved by inclusion of mesityl protecting groups [27]. Extension of the BFC model with thiophene provides further interesting materials [28]. A reasonable corollary to this behavior would assert that derivatives of TBF and pentabenzocorannulene would produce air-stable radical and ionic PAHs, and that coupling such fragments would lead to stable redox-active carbon sheets.

## Conclusion

In conclusion, the “surprise” in the surprising p*K*_a_ for BIC and FIC was in our expectation of the deprotonated forms as poorly delocalized fluorenyl anions. The CL perspective provided us a different way to look at these compounds and interpret them as phenyl-substituted CpHs, thus reconciling their higher acidity. That perspective led us to a CL graph representation that predicted/rationalized additional acidic CpH-PAHs and sharpened our understanding of an “azacorannulene”. Working from graph structures based on chemically stable (“group elementary”) nodes is a useful principle in molecular design and chemical synthesis. Such a perspective is important to understand fundamental physical organic molecular properties as well as to predict desirable unforeseen new material designs (e.g., redox-active electrooptical materials).

## Methods

### p*K*_a_ measurements in acetonitrile

The experimental setup and methodology for the p*K*_a_ determination of BFC and FIC in acetonitrile was essentially the same as described in detail in previous papers [12–13]. A brief description will follow.

The p*K*_a_ determinations in acetonitrile are based on the determinations of differences of p*K*_a_ values of two acids. In this case one compound is a reference acid with a previously known p*K*_a_ value and the other acid is either FIC or BFC. Both compounds, as well as the references are also separately titrated in order to obtain the UV–vis spectra of the acids in neutral as well as in deprotonated forms.

Then, the same titration is done with a mixture of measured acid (FIC or BFC) and a reference acid. Using the spectral data from the titrations of mixtures the dissociation levels α = [A^−^]/([A^−^] + [HA]) of both acids in all the mixtures formed during titration are calculated and are then in turn used to calculate the differences of p*K*_a_ values (∆p*K*_a_) of FIC or BFC and the used reference acids according the following equation:




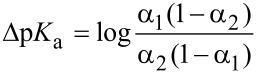




The p*K*_a_ values of BFC and FIC are estimated as a result of ∆p*K*_a_ measurements against different reference acids.

A Perkin-Elmer Lambda 40 UV–vis spectrophotometer connected with optical fibre cables to an external cell compartment inside a MBraun Unilab glovebox filled with 99.999% pure argon was used for the spectrophotometric titrations. This setup ensured that during all titrations the moisture and oxygen contents in argon were always under 10 ppm during measurements. Triflic acid (Aldrich, 99+%) and phosphazene base P_2_-Et (165535-45-5, Aldrich, ≥98%) were used to prepare the acidic and basic titrant solutions, respectively. Acetonitrile (Romil 190 SpS far UV/gradient quality) was used as solvent after drying with molecular sieves (3 Å), which lowered the water content to a range of 2–6 ppm.

Acids with previously published p*K*_a_ values in acetonitrile were used as reference acids [12–13]. Usually this kind of ∆p*K*_a_ measurements are done against three reference acids but due to the lack of suitable reference acids in the weakly acidic p*K*_a_ region in acetonitrile only two reference acids were used. The results are presented in Table 2. We estimate the standard uncertainties of the assigned p*K*_a_ values of BFC and FIC as 0.15 p*K*_a_ units.

**Table 2:** Results of p*K*_a_ measurements in acetonitrile.

Acid	Reference acid	p*K*_a_ (Ref)	Δp*K*_a_	p*K*_a_ (acid)	assigned p*K*_a_

BFC	9-C_6_F_5_-fluorene	28.14	0.55	27.59	27.6
	(4-Me-C_6_F_4_)(C_6_H_5_)CHCN	26.98	−0.78	27.76	

FIC	9-C_6_F_5_-fluorene	28.14	0.38	27.76	27.8
	(4-Me-C_6_F_4_)(C_6_H_5_)CHCN	26.98	−0.94	27.92	

### p*K*_a_ predictions using correlation analysis

The p*K*_a_ values of fluorene, indene, cyclopentadiene, 9-phenylfluorene, and diphenylmethane in acetonitrile were estimated by averaging the values obtained from their experimental p*K*_a_ values in DMSO [5] and three correlation equations: equations 2.1 and 2.2 in Ref. [13] and correlation equation composed of experimental p*K*_a_ values of 9-C_6_F_5_-fluorene, octafluorofluorene, fluoradene, (4-Me-C_6_F_4_)(C_6_H_5_)CHCN, (C_6_F_5_)(C_6_H_5_)CHCN, 9-COOMe-fluorene, and 9-CN-fluorene in acetonitrile [12–13] and DMSO [5]. All these p*K*_a_ estimations involve significant extrapolation and the agreement between the estimates obtained from different equations is not good. Therefore, the standard uncertainties of these obtained p*K*_a_ estimates are high: 2 p*K*_a_ units for fluorene, indene and 9-phenylfluorene, 3 p*K*_a_ units for cyclopentadiene (as this acid is very different from the acids used for developing the correlation equations), and 3 p*K*_a_ units for diphenylmethane (as very strong extrapolation is involved with this acid) [12–13].

The p*K*_a_ values of FIC and BFC in DMSO were estimated from the experimental p*K*_a_ values in acetonitrile determined in this study using the average values from the same three equations as above, however, using them in reverse. The standard uncertainty estimates of the p*K*_a_ values of FIC and BFC in DMSO are 0.8 p*K*_a_ units.

### Synthesis

Material used to measure the acidity of compounds BFC and FIC was prepared previously by methods reported in reference [9].

### Computational methods

The structural and energetic analyses of the molecular systems for all compounds described in this study were carried out with the B97-D dispersion enabled density functional method [29–30], using an ultrafine grid, together with the def2-TZVPP basis set [31]. Full geometry optimizations were performed and uniquely characterized via second derivatives (Hessian) analysis to establish stationary points and effects of zero point and thermal energy contributions. Effects of solvent employed the COSMO:ab initio continuum method [32–33], using a dielectric as in experiment, and fully optimized using radii of Klamt [34]. Electronic and thermal free energy differences between neutral and anion were compared to a reference pentafluorophenylfluorene, and subsequently converted to p*K*_a_ values by dividing by 1.36. Visualization and analysis of structural and property results were obtained using Avogadro [35]. Our group develops GAMESS [36] and has also contributed to Gaussian software packages, in this work the G09 ES64L-G09RevE.01 version [37] of the latter was used.

## Data Availability

All data that supports the findings of this study is available in the published article and/or the supporting information of this article.
